# Supplementary material for: An application of YOLOv8 integrated with attention mechanisms for detection of grape leaf black rot spots
Source: PLoS One. 2025 Apr 15;20(4):e0321788. doi: 10.1371/journal.pone.0321788 (PMC11999144; doi:10.1371/journal.pone.0321788)
Supplement: Attachment — Submitted filename: point-by-point response to reviewers.docx [file pone.0321788.s001.docx]

**Reviewer #1**

**1.no furture work**

Thank you for your suggestions. Based on your advice, we have added information about the direction of future research in the introduction section. We hope to receive your approval.

**2.what are the challenges faced**

Thank you for your suggestions. Based on your advice, we have added information about the direction of future research in the introduction section. We hope to receive your approval. We have expanded the content in Section 4 by adding two new subsections: 4.1 "Previous Research" and 4.5 "Analysis of Test Results in Natural Environments."

**3.the work is well done and experimented, the imporved method result is okay**

Thank you for your acknowledgment. We will continue to follow up on the subsequent research.

**4. the gramatical errors was avoided**

We have reviewed the entire text based on your suggestions. Additionally, we have invited a professional grammar teacher to further refine the manuscript. You will be able to see the improvements in our newly submitted version.
**5. similaruty test shows that it is 10-11% which is okay**

Thank you for your acknowledgment. We will continue to follow up on the subsequent research.

**Reviewer #2**

1- **The introduction did not define the work environment of the research field, nor did it address its general basics or the research gap it suffers from.**

Thank you very much for your valuable feedback. Based on your guidance, we have thoroughly revised the introduction section. We have included a detailed description of the main work of this paper, the current challenges encountered, and the future research directions of our research group. You will notice these additions in our newly submitted manuscript. We hope our modifications meet with your approval.
**2- Add a paragraph at the end of the introduction that indicates the structure of the research with all its parts.**

Thank you very much for your invaluable suggestions. Following your guidance, we have provided an explanation in the introduction section under "The main work of this paper is as follows." The focus of this paper lies in improving the algorithm while comparing it with the YOLO system algorithm. Additionally, we have expanded the content in Section 4 by incorporating two new subsections: 4.1 "Previous Research" and 4.5 "Analysis of Test Results in Natural Environments."
**3- Refer to the dataset used with a reference that contains it and add it to the references.**

Thank you for your suggestion. We have added the citation for the dataset in the References section. We hope this meets with your approval.
**4- Explaining the difference between CNN, and Spatial Pyramid Dilated Convolution**

Thank you for your inquiry. We have made extensive modifications in the section titled "Grape Leaf Spot Detection Based on Improved YOLOv8." In the newly submitted manuscript, we have provided a more detailed elaboration, which includes addressing your concerns and re-explaining the workflow and detailed steps of the network. We hope the improved manuscript will meet with your approval.
**5- Clarify future work on the presented work and expand conclusions based on a well-constructed discussion of the numerical values of the results in tables and figures.**

This issue certainly warrants our attention. Our research has consistently focused on disease detection. In the revised manuscript, we have included our previous related work titled "Grape Leaf Black Rot Detection Based on Super-Resolution Image Enhancement and Deep Learning." Additionally, we have added Section 4.5, "Analysis of Test Results in Natural Environments." To further evaluate the advantages of the improved network, we have introduced the "PT" metric. In Table 3, we have also conducted further discussions and analyses. We hope the enhanced manuscript will meet with your approval.
**6- Include additional references published in the years 2023-2024**
 Thank you for your feedback. We have made revisions to the manuscript. As you will see in the newly submitted version, the citations in the introduction section have been reorganized. We have included recent related research and data analysis. We hope the revised manuscript meets with your approval.
